# Ethics in mental health research

**DOI:** 10.1192/j.eurpsy.2023.203

**Published:** 2023-07-19

**Authors:** M. Pinto da Costa

**Affiliations:** Institute of Psychiatry, Psychology & Neuroscience, King’s College London, London, United Kingdom; Institute of Biomedical Sciences Abel Salazar, University of Porto, Porto, Portugal

## Abstract

**Abstract:**

The boundaries between innovative clinical practices and research-related experimentation can be difficult to distinguish. Whilst mental health research has increased remarkably over recent decades, ethics is required to maintain and improve the standards of mental health research. Ethics and science can be seen as opposing forces with different aims championed by different people. However, ethics should not be a barrier to scientific advancement, but rather as the way that mental health research can be conducted with broader societal support, with the expectation of bringing wider benefit to people with mental illness. Scientific advancements may also lead to novel ethical problems and raise questions in relation to oversight responsibilities. Mental health research faces several unique ethical challenges. This presentation will provide an overview of the ethical issues linked with mental health research.

**Disclosure of Interest:**

None Declared

